# Do epinephrine auto-injectors have an unsuitable needle length in children and adolescents at risk for anaphylaxis from food allergy?

**DOI:** 10.1186/s13223-016-0110-8

**Published:** 2016-03-06

**Authors:** Sten Dreborg, Xia Wen, Laura Kim, Gina Tsai, Immaculate Nevis, Ryan Potts, Jack Chiu, Arunmozhi Dominic, Harold Kim

**Affiliations:** Women’s and Children’s Health, University of Uppsala, Uppsala, Sweden; Faculty of Science, McGill University, Montreal, Canada; Faculty of Medicine, University British Columbia, Vancouver, Canada; Goodman School of Business, Brock University, St. Catharines, Canada; Farncombe Family Digestive Health Unit, McMaster University, Hamilton, Canada; Department of Medicine, Western University, London, Canada; Department of Medicine, McMaster University, Hamilton, ON Canada

**Keywords:** Anaphylaxis, Food allergy, Epinephrine, Epinephrine auto-injector, Allergy, Skin to bone distance, Skin to muscle distance, Intramuscular, Subcutaneous, Epimysium

## Abstract

**Background:**

Food allergy is the most common cause of anaphylaxis in children. Intramuscular delivery of epinephrine auto-injectors (EAI) is the standard of care for the treatment of anaphylaxis. We examined if children and adolescents at risk of anaphylaxis weighing 15–30 kg and >30 kg would receive epinephrine into the intramuscular space with the currently available EAI in North America and Europe.

**Methods:**

The distance from skin to muscle (STMD) and skin to bone (STBD) on the mid third anterolateral area of the right thigh was measured by ultrasound applying either high pressure (_max_) or slight pressure (_min_) in 102 children weighing 15–30 kg (group 1) and 100 children and adolescents, weighing more than 30 kg (group 2).

**Results:**

Using a high pressure EAI (HPEAI), Epipen Jr^®^ and Auvi-Q^®^/Allerject^®^ 0.15 mg, 11/102 (11 %) children in group 1 and 38/102 (38 %) using another HPEAI, Jext^®^, had a STMD_max_ that showed a risk of intraosseous injection. There was a 1 % risk of subcutaneous injection with these devices. There was no risk of intraosseous injection using a low pressure EAI (LPEAI), Emerade^®^. In group 2, the risk of intraosseous injection using a HPEAI was 3 % and no risk using a LPEAI. However, the risk of subcutaneous injection using HPEAI was 9 % and using LPEAI was 2 %.

**Conclusion:**

There is a risk of intraosseous injection using HPEAI (Epipen^®^/Epipen Jr^®^, Auvi-Q^®^/Allerject^®^ and especially Jext^®^) in children at risk of anaphylaxis. There was also a risk of subcutaneous injection using the currently available HPEAI in children and adolescents.

**Electronic supplementary material:**

The online version of this article (doi:10.1186/s13223-016-0110-8) contains supplementary material, which is available to authorized users.

## Background

Anaphylaxis is a potentially life-threatening reaction that can occur from a number of different triggers. In children, food allergy is the most common identified cause of anaphylaxis [1]. The true prevalence of food allergy in children is difficult to define because in most studies double blind placebo controlled food challenges are not performed to properly confirm the diagnosis [2]. Recent estimates suggest a prevalence of approximately 6 % in young children and 4 % in adults [1–3]. Currently, the prompt administration of intramuscular epinephrine is the recommended treatment for anaphylaxis [1, 2]. Outside of the hospital setting, epinephrine auto-injectors (EAI) are the standard method of administering epinephrine. In North America, the Epipen^®^ [4] and Auvi-Q^®^ [5] (Allerject^®^ in Canada [6]) are currently the most commonly prescribed EAI. Importantly, there are different EAI available in Europe with different physical characteristics including pressure required for delivery of the medication and needle lengths Table 1.

**Table 1:** Characteristics for epinephrine auto-injectors available in Europe and North America

Epinephrine autoinjector (EAI)	Epinephrine dose	Needle length(mm)		Pressure against the thigh
		Exposed needle length	Distance skin to muscle	
Epipen Jr^®^, Auvi-Q^®^/Allerject^®^	0.15 mg	12.7	10.7	Thrust/Pressed firmly
Epipen^®^Auvi-Q^®^/Allerject^®^	0.3 mg	15.2	13.2	
Jext^®^ 0.15 mg	0.15 mg	15.7	13.7	Pressed firmly
Jext^®^ 0.3 mg	0.3 mg	15.7	13.7	
Emerade^®^ 0.15 mg	0.15 mg	16	14	Slight pressure
Emerade^®^ 0.3 mg	0.3 mg	23	21	
Emerade^®^ 0.5 mg	0.5 mg	23	21	

The Epipen Jr^®^ and the Auvi-Q^®^/Allerject^®^ 0.15 mg are officially indicated for pediatric patients at risk of anaphylaxis who weigh between 15 and 30 kg. In Europe, Jext^®^ and Emerade^®^ are available with 0.15 mg per dose. For patients who weigh >30 kg, Epipen^®^ and Auvi-Q^®^/Allerject^®^ 0.30 mg are indicated. In Europe, Jext and Emerade^®^ are available at the same strengths. Emerade^®^ is also available at a 0.5 mg dose indicated for treatment of adults. The needle lengths and other properties of EAI are given in Table 1 [4–6]. There has been concern that the EAI may not deliver epinephrine to the intramuscular space in both adults and children. In both adults and children with and without risk of anaphylaxis, it has been identified that a significant number of patients may receive epinephrine subcutaneously with the currently available EAI [7–10]. There has been a contradictory study showing that in children weighing <15 kg, with confirmed food allergy, a significant number of children may be at risk of receiving the injection into the periosteum or bone [11].

In this present study, we sought to evaluate whether children and adolescents who are at risk of anaphylaxis from food allergy weighing 15–30 kg and >30 kg would receive epinephrine into the intramuscular space, the bone or subcutaneous space using currently available EAI in North America and Europe.

## Patients and methods

### Patients

All of the parents or legal guardians of each patient provided written, informed consent before participating in this study. The study was approved by the Lawson Health Research Institute Ethics Board at the Western University in London, Ontario, Canada.

Consecutive patients who were less than 18 years of age, with diagnosed food allergy based on an appropriate history and positive skin prick testing were recruited into the study. They were separated into two groups: those who weighed between 15 and 30 kg, Group 1 (n = 102), and those who weighed more than 30 kg, Group 2 (n = 100). Patient characteristics are given in Table 2. All of these subjects required a prescription for an EAI for food allergy. There were no clinical exclusion criteria. The subjects were assessed from July 2012 to June 2014.

**Table 2:** Clinical characteristics of children in the study

Character	Group 1 15–30 kg mean (range) n = 102	Group 2 >30 kg mean (range) n = 100	Difference p<
Age (years)	5.7 (2–10)	13.3 (7–18)	–
Boys/girls	62/40	60/40	ns
Height (cm)	115.8 (83.8–144.8)	161.7 (121.9–190.5)	–
Weight (kg)	21.3 (14.5–29.9)	54.4 (30.4–88.9)	–
BMI^a^ (kg/m^2^)	15.9 (10.8–24.3)	20.5(14.5–31.4)	0.00001

All subjects had food allergy necessitating the prescription of EAI

^a^two-tailed t-test

– not meaningful comparison

*ns* not significant

### Ultrasound estimation distance skin to muscle (STMD) and skin to bone (STBD)

An ultrasound of the anterolateral aspect of the right mid-thigh, i.e., the recommended location for injecting an EAI, was completed on all of the subjects with and without pressure using a Sonosite Titan^®^ ultrasound machine. The pressure was applied to simulate the pressure required to trigger the HPEAI. The estimated pressure to mimic the pressure applied by the Epipen Jr^®^, Epipen^®^, Auvi-Q^®^/Allerject^®^ and Jext^®^ was 8 pounds [9]. To mimic the pressure applied to Emerade^®^, minimal pressure was applied.

**Fig. 1:**
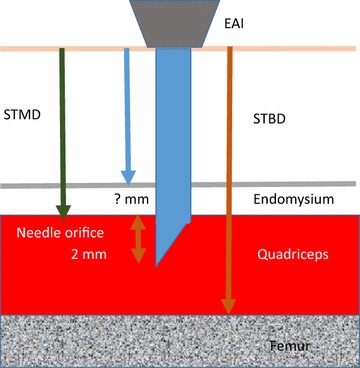
Novel approach for measuring the distance from skin to bone and skin to muscle. To obtain an intramuscular injection, the STMD must be at the most the exposed needle length minus 2 mm (needle orifice). There is a risk of intraosseous injection if the STBD is less than the full exposed length of the needle (also see Table 1)

Four ultrasound measurements were collected on each patient: skin-to-muscle depth with low pressure (STMD_min_), skin-to-muscle depth with “maximal” pressure (STMD_max_), skin-to-bone depth with minimal pressure (STBD_min_) and skin-to-bone depth with “maximal” pressure (STBD_max_). Figure 1 shows the principle of measurement. The STMD was measured from the skin surface to the inner aspect of the fascia of the vastus lateralis muscle and the bone depth was measured at the outer aspect of the periosteum of the femur. The ultrasound measurements were performed by one investigator.

### Analysis

The two different weight groups were analyzed separately. Recently, Diacono et al. [9] found the needle must pass not only through the fascia lata but also the epimysium to deliver a drug into the intramuscular space. If part of the orifice stays within the epimysium during the injection, epinephrine will spread within the loose epimysium tissue. Therefore, the whole needle orifice must pass into the muscle for proper administration. The needle lengths used are those given by the manufacturers. The ultrasounds of the mid-anterolateral thigh with and without pressure were measured to identify whether children with prescribed EAI would be at risk of subcutaneous or periosteal/intraosseous delivery of epinephrine. The length of the needle orifice was estimated to be 2 mm. This length was used as our measurement of the needle orifice distance for the Epipen Jr^®^ was just over 2 mm and the Emerade^®^ measurement is 1.7 mm (information supplied by the manufacturer Medeca Pharma). Therefore, we used the needle length minus 2 mm for estimation of the STMD threshold. For estimation of the STBD the full length of the needles were used (Table 1 and Fig. 1).

### Outcome variables

We used two primary outcome variables: The proportion of children with (1) STBD less than the needle length (2) STMD more than the needle length minus 2 mm (Table 1 and Fig. 1).

### Statistics

Differences between groups were calculated using two-tailed t-test. Correlations were calculated using Spearman’s rank correlation coefficient. Also, we estimated the proportion of subjects who would likely receive epinephrine intramuscularly with the different EAI available in North America and Europe.

## Results

The patient characteristics for both group 1 (15–30 kg) and group 2 (>30 kg) are presented in Table 2. The BMI of group 1 was significantly less than that of group 2 (p < 0.00001) (Table 2). Only 13/202 children had a BMI exceeding 25 kg/m^2^. Therefore, the weight increased with age but only a few teenagers were overweight or obese.

In Table 3, further characteristics are analyzed for children in Group 1 and differences between those with a STBD_max_ ≥ 12.7 mm and those with a STBD_max_ < 12.7 mm, i.e., those without (n = 91) and those with (n = 11) a risk of intraosseous injection using a HPEIA (Epipen Jr^®^ and Auvi-Q^®^/Allerject^®^ 0.15 mg). Since the age of children varied (interquartile range 48 and 84 months), and the weight within the range 15–30 kg, all other data will vary, most for the STBD, p < 0.0002 and BMI, p = 0.0012.

**Table 3:** Characteristics of children 15–30 kg

Characteristics	Total (n = 102)	Patients with STBDmax		t test p value
		≥12.7 mm (n = 91)	<12.7 mm (n = 11)	
Age (months) median (IQR)	72 (48–84)	72 (48–84)	60 (48–78)	0.019
Males. n (%)	62 (60.4)	54 (59.3)	8 (72.3)	0.074
Weight (kg). mean (SD)	21.3 (4.0)	21.4 (3.9)	19.7 (4.2)	ns
Height (m). mean (SD)	1.16 (0.12)	1.16 (0.12)	1.18 (0.15)	ns
BMI (kg/m^2^). mean (SD)	15.8 (2.0)	16.1 (1.9)	14 (1.4)	0.0012
STMD_max_ (mm). mean(SD)	5.8 (1.5)	5.9 (1.5)	4.8 (1.0)	0.025
STMD_min_ (mm). mean (SD)	6.7 (1.7)	6.8 (1.7)	5.5 (1.3)	0.019
STBD_max_ (mm). mean (SD)	16.7 (3.0)	17.3 (2.5)	11.6 (0.9)	<0.000001
STBD_min_ (mm). mean (SD)	29.5 (3.9)	30.0 (3.7)	25.4 (2.6)	0.0002

Distance skin surface to bone with high pressure applied to the ultrasound probe (STBD_max_). Data on one needle length 12.7 mm are given. Those with STBD_max_ < 12.7 mm. i.e., with a risk of penetrating the femur and those with more than 12.7 mm STBD_max_. The differences between groups was tested by a two-tailed t-test. The inter-quartile ratio (IQR) percentage (%) and standard deviations (SD) are given within the brackets

In Table 4, further characteristics of children are analyzed, i.e., among adolescents in group 2 and differences between those with a STBD_max_ ≥ 15.2 mm and those with a STBD_max_ < 15.2 mm i.e., those without (n = 97) and those with (n = 3) a risk of intraosseous injection using a HPEAI (Epipen^®^ and Auvi-Q^®^/Allerject^®^ 0.3 mg). Since the age of children varied (interquartile range 144 and 192 months), and the weight between 30 and 89 kg, all other data varied, especially for the STBD, p = 0.022.

**Table 4:** Characteristics of children and adolescents > 30 kg

Characteristics	Total (n = 100)	Patients with STBDmax		t test p value
		≥15.2 mm (n = 97)	<15.2 mm (n = 3)	
Age (months). median (IQR)	168 (144–192)	168 (144–192)	144 (114–144)	0.07
Males. n (%)	60 (60)	57 (58.8)	3 (100)	ns
Weight (kg). mean (SD)	54.5 (15.4)	55 (15.3)	36.4 (6.0)	0.012
Height (m). mean (SD)	1.62 (0.14)	1.62 (0.14)	1.41 (0.03)	0.041
BMI (kg/m^2^). mean (SD)	20.5 (3.9)	20.5 (3.9)	18.4 (3.9)	ns
STMD_max_ (mm). mean (SD)	7.4 (3.8)	7.5 (3.8)	4.6 (0.1)	ns
STMD_min_ (mm). mean (SD)	8.4 (4.4)	8.5 (4.5)	5.4 (0.4)	ns
STBD_max_ (mm). mean (SD)	25.5 (7.9)	25.8 (7.7)	13.5 (0.5)	0.0075
STBD_min_ (mm). mean (SD)	41.7 (8.9)	42.0 (8.8)	30.1 (4.8)	0.022

Data on one needle length of 15.2 mm are given. Those with STBD_max_ < 15.2 mm. i.e., at risk of penetrating the femur and those with >15.2 mm STBD_max_. The differences between groups was tested by a two-tailed t-test. The inter-quartile ratio (IQR) percentage (%) and standard deviations (SD) are given within brackets

No child had a risk of intraosseous or subcutaneous injection using a LPEAI, Emerade^®^.

### Group 1 (15–30 kg)

In group 1, there were 11/102 subjects with a STBD_max_ < 12.7 mm with a risk of intraosseous injection and 1/102 subject with STMD_max_ > 10.7 mm (Epipen Jr^®^ and Auvi-Q^®^/Allerject^®^ 0.15 mg) when using Epipen Jr^®^/Auvi-Q^®^/Allerject^®^, with a risk of subcutaneous injection of epinephrine (Table 3).

Furthermore, STBD_max_ was < 15.7 mm (Jext^®^) in 38/102 children, one child had a STMD_max_ > 10.7 mm, no child had STBD_min_ < 16 mm (Emerade^®^) and no child had a STMD_min_ < 14 mm (Table 5).

**Table 5:** Number of children 15–30 kg at risk of subcutaneous injection (STMD_max_ and STMD_min_) or periosteal or bone injection (STBD_max_ and STBD_min_)

Brand	Needle length mm	STMD_min_	STMD_max_	STBD _min_	STBD _max_
EpipenJr^®^ 0.15 mg Auvi-Q^®^ 0.15 mg					
STBD_max_	12.7	–	–	–	11
STMD_max_	10.7	–	1	–	–
Jext^**®**^					
STBD_max_	15.7	–	–	–	38
STMD_max_	13.7	–	0	–	–
Emerade^®^ 0.15 mg					
STBD_min_	16.0	–	–	0	–
STMD_min_	14.0	0	–	–	–

Number of children at risk of getting a subcutaneous injection (STMD_max_ and STMD_min_) or injection into the periosteum or bone (STBD_max_ and STBD_min_) for the different EAIs. The data are in percentages. For estimation of the penetration of the needle into the muscle, STMD, the needle length has been reduced by 2 mm. i.e., the length of the needle’s orifice. The thickness of the endomysium has been neglected

In summary, 11/102 children using a HPEAI, Epipen Jr^®^ or Auvi-Q^®^/Allerject^®^, and 38/102 children, if using Jext^®^, would have the risk of an injection in the bone. One of the 102 children would have the risk of a subcutaneous injection. No patients were at risk of an intraosseous or subcutaneous injection using a LPEAI, Emerade^®^ device.

### Group 2 (>30 kg)

For the >30 kg group, using Epipen^®^, Auvi-Q^®^/Allerject^®^ or Jext^®^ there was 3/100 with STBD_max_ < 15.2 mm indicating a risk of injecting into bone. Furthermore, 9/102 children had a STMD_max_ > 13.2 mm and >13.7 mm suggesting a risk of subcutaneous injection (Table 6).

**Table 6:** Number of children and adolescents >30 kg at risk of subcutaneous injection (STMD_max_ and STMD_min_) or periosteal or bone injection (STBD_max_ and STBD_min_)

EAI	Needle length mm	STMD_min_	STMD_max_	STBD_min_	STBD_max_
Epipen^®^ 0.3 mg Auvi-Q^®^ 0.3 mg					
STBD_max_	15.2	–	–	–	3
STMD_max_	13.2	–	9	–	–
Jext^®^ 0.3 mg					
STBD_max_	15.7	–	–	–	3
STMD_max_	13.7	–	9	–	–
Emerade^®^ 0.3 and 0.5 mg					
STBD_min_	23.0	–	–	0	–
STMD_min_	21.0	2	–	–	–

Number of children at risk of getting a subcutaneous injection (STMD_max_ and STMD_min_) or injection into the periosteum or bone (STBD_max_ and STBD_min_) for the different EAIs. The data are in percentages. For estimation of the penetration of the needle into the muscle, STMD, the needle length has been reduced by 2 mm. i.e., the length of the needle’s orifice. The thickness of the endomysium has been neglected

Among the 100 children weighing >30 kg, no child had a STBD_min_ < 23 mm and 2/100 had a STMD_min_ > 21 mm indicating two children would be at risk getting a subcutaneous injection with a low pressure EAI such as Emerade^®^.

In summary, 3/100 children would have a risk of an injection into the bone and 9/100 an injection in the subcutaneous tissue when using the HPEAI (Epipen^®^, Auvi-Q^®^/Allerject^®^ or Jext^®^), whereas 2/100 patients had a risk of a subcutaneous injection using a LPEAI (Emerade^®^).

### Correlations

The strongest correlation was found between STMD_max_ and STMD_min_ when all children were considered (r = 0.96) (Table 7). This correlation was also present in both groups, with group 1, r = 0.92, and group 2, r = 0.96 (p < 0.05). The STBD_max_ and STBD_min_ correlated significantly (p < 0.05) in all three groups, with a correlation coefficient 0.89, 0.56 and 0.88 respectively (Tables 7, 8 and 9).

**Table 7:**
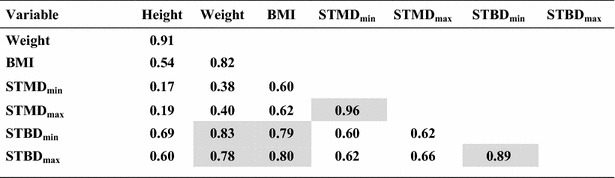
Correlations for children >15 kg and <18 years (n = 202)

Bold figures indicate significant results at p < 0.05 level. Shadowed values are highlighting the strongest correlations (r > 0.75) that may be of importance

**Table 8:**
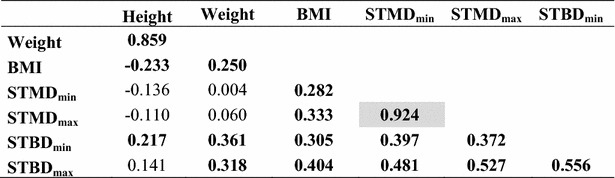
Correlations for children 15–30 kg (n = 102)

Bold figures indicate significance at p < 0.05 level. Shadowed values are highlighting the strongest correlations (r > 0.75) that may be of importance

**Table 9:**
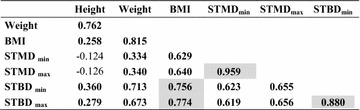
Correlations for children weighing >30 kg and <18 yrs

Bold figures indicate significance at the p < 0.05 level. Shadowed values are highlighting the strongest correlations (r > 0.75) that may be of importance

**Fig. 2:**
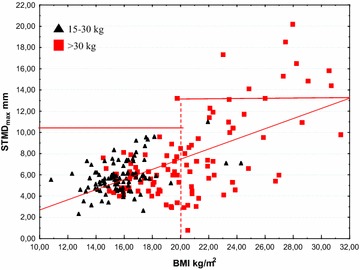
STMD_max_ versus BMI. The *horizontal lines* indicate the length of the needle of Epipen Jr^®^ and Epipen^®^ respectively, both minus 2 mm, the estimated distance from the point of the needle to the upper limit of the orifice. The *vertical dotted line* indicates the proposed limit using Epipen^®^ to avoid subcutaneous injection. The nine subjects at risk of subcutaneous injection using Epipen^®^ were all overweight. However, children with a BMI < 20 kg/m^2^ are not at risk getting a subcutaneous injection when using the EpiPen^®^ needle 15.2 mm

**Fig. 3:**
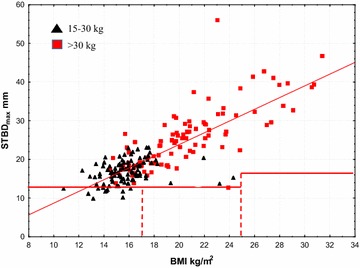
STBD_max_ versus BMI. The *horizontal lines* indicate the full length of the needle of Epipen Jr^®^ and Epipen^®^ respectively. The *vertical dotted lines* indicate those at risk of intraosseous injection using Epipen Jr^®^ are those with a BMI < 17 kg/m^2^ and those at risk of intraosseous injection using Epipen^®^ are those with a BMI < 25 kg/m^2^

When all children were considered the BMI correlated with STMD_min_ (r = 0.60), STMD_max_ (r = 0.62), STBD_min_ (r = 0.79) and STBD_max_ (r = 0.80), all p < 0.05 (Table 7). Even in the two separate groups, BMI correlated with the STMD and STBD measures, but at a lower level (Table 8 and 9). In group 1, there was no risk of subcutaneous injection for a BMI < 20 kg/m^2^, but there is a risk of intraosseous injection for a BMI < 25 kg/m^2^ using the Epipen Jr^®^ needle length. In group 2, there was a risk of subcutaneous injection for a BMI > 20 kg/m^2^ and there was no risk of intraosseous injection for BMI > 25 kg/m^2^ using the Epipen^®^ needle length (Figs. 2 and 3).

Weight, the variable typically used for dosing epinephrine, correlated with the primary variable, BMI (r = 0.85, 0.25 and 0.82 respectively (Tables 7, 8 and 9), and STBD_min_ (r = 0.83, 0.36 and 0.71, respectively).

### Characteristics of children at risk of intraosseous injection

In group 1, data on the 11 patients with a STBD_max_ < 12.7 mm (EpipenJr^®^ or Auvi-Q^®^/Allerject^®^) (Additional file 1: Table S1a) and the data on the 38 patients with a STBD_max_ < 15.2 mm (Jext^®^) (Additional file 1: Table S1b) are shown.

Data on three children from group 2 with a STBD_max_ < 15.2 mm (Epipen^®^ or Auvi-Q^®^/Allerject^®^ 0.3 mg), i.e., with a risk of intraosseous injection, are shown in Additional file 1: Table S1c and data on the nine patients with a STMD_max_ > 15.2 mm (Epipen^®^ or Auvi-Q^®^/Allerject^®^ 0.3 mg) are shown in Additional file 1: Table S1e.

### Compression of subcutaneous tissue, muscle and total tissue

Using data from all 202 children, the subcutaneous tissue was compressed a mean of 1.1 mm, quartiles 0.4–1.3 mm, the muscle mean of 12.7 mm, quartiles 9.4–15.8 mm, and the total compression mean of 13.8 mm, quartiles 10.8–16.8 mm. Thus, the compression originated mainly from the muscle compartment, mean of 91.3 %, quartiles 8.8–96.4 %.

## Discussion

This is the first study assessing whether EAIs would deliver epinephrine into the intramuscular space in children with food allergy who are in the proper weight indications for the Epipen^®^ and Auvi-Q^®^/Allerject^®^. As well, we assess whether EAI only available in Europe would be different than the Epipen^®^ and Auvi-Q^®^/Allerject^®^. There is one study assessing STMD without pressure in children who were not at risk of anaphylaxis. In that study, Stecher [7] found that 12 % of children weighing less than 30 kg were at risk of subcutaneous injections. We found that more children weighing 15–30 kg were at risk of intraosseous injection (38 %) using a HPEAI not commonly used in North America (Jext^®^).

Importantly, Diacono et al. [9] showed that drug delivery into the muscle requires that the whole opening of the needle bevel must penetrate completely through the muscle fascia. This current study is the first using a modified needle length threshold, which takes into account the likelihood that the whole needle bevel should penetrate through the whole muscle fascia in order to deliver the drug into the intramuscular space. In this study, we used 2 mm less than the total needle length to predict whether epinephrine would be delivered intramuscularly. However, we used the true exposed needle length to predict whether the needle would hit the bone. We believe this approach would be the most conservative method to predict proper injection into the muscle. As well, the strongest correlations were found between STMD_min_ and STMD_max_. This observation may be interpreted that the pressure on the probe when measuring STMD_max_ was consistent.

This study showed that children weighing 15–30 kg would be at 11 % risk of the EIA needle reaching the femur with the Epipen Jr^®^ or Auvi-Q^®^/Allerject^®^ 0.15 mg and 38 % with Jext^®^. In children weighing >30 kg, 3 % of children were at risk injection into bone with Jext. There was no risk of injection into bone using the low pressure EAI, Emerade^®^. Conversely, 1 % of children weighing 15–30 kg and 9 % of those weighing >30 kg using Epipen Jr^®^, Auvi-Q^®^/Allerject^®^ or Jext^®^ may receive the epinephrine subcutaneously. No children at 15–30 kg and 2 % of those weighing >30 kg would have a subcutaneous injection using the low pressure EAI, Emerade^®^. We feel these findings are clinically relevant as the EAI have a significant risk of delivering the epinephrine into an inappropriate tissue compartment leading to unpredictable serum levels of epinephrine. Furthermore, the risk of inappropriate tissue delivery varied with the different EAI. The risk varied due to different exposed needle lengths and the force required to trigger the devices.

Currently, there are no clinical studies proving that intramuscular, subcutaneous and interosseous injections of epinephrine would lead to different clinical outcomes. However, there are small controlled studies showing that intramuscular epinephrine likely leads to higher and more rapid peak epinephrine levels. There is one study in adults [12] and one study in children [13]. The adult study has some potential limitations. It was a “prospective, randomized, blinded, placebo-controlled, 6-way crossover study of intramuscular versus subcutaneous injection of epinephrine in young men.” The study included only 13 male subjects. The mean BMI, 36.6 ± 4.6 [range 20–64] indicated there was very likely increased subcutaneous fat layer in some individuals. There appears to be an early peak in serum epinephrine levels with a later peak in epinephrine levels at 45 min. The intramuscular injections were given with Epipen^®^. This is a high pressure EAI with a relatively short needle (15.2 mm). Thus, we propose that, in some subjects, the epinephrine was deposited subcutaneously leading to this second peak.

In the pediatric study by Simons [13], again the numbers are small with only nine children in the group receiving epinephrine intramuscularly (by Epipen^®^). There appears to be a secondary peak of serum epinephrine in this study as well. Interestingly, a study comparing Epipen^®^ with Auvi-Q^®^/Allerject^®^ [14] showed these products were similar in terms of achieving serum epinephrine levels. However, again there appears to be an early peak of epinephrine and a secondary peak at 30 min. We believe this second peak could have been eliminated if all of the subjects had confirmed intramuscular injections of epinephrine.

Although there are no human studies assessing clinical effect or epinephrine levels with periosteal or intraosseous injections of epinephrine, studies suggest that intraosseous injection has similar effects to intravenous infusion [15]. Therefore, intraosseous injection with the epinephrine from EAIs could lead to dangerously high levels in children. There is recent evidence showing that intravenous bolus of epinephrine leads to an increased risk of cardiovascular side effects compared to intramuscular injection [16]. As well, we believe the EAIs would penetrate through the bone in children if enough pressure is applied as shown in a case report where an EAI needle was inadvertently injected completely through an adult’s finger [17]. But in older children, it is possible that the EAI needles would not penetrate through the cortical bone of the femur.

There are some limitations to this study. Firstly, this study was completed at one clinical site and one non-blinded physician completed all of the ultrasound measurements. The ultrasound measurements were easy to perform and a second physician (a radiologist) confirmed the measurements in 17 randomly selected children showing no significant difference between the two investigators. The patients were all from southwestern Ontario, Canada. Therefore, this population may not be truly representative of other populations. As well, the pressure applied, to mimic the pressure applied to a high pressure EAI, was estimated to be 8 pounds, but it was not measured with each measurement. It would be optimal to complete the measurements with a consistently applied pressure with an ultrasound probe with the exact surface area of each of the EAI. But this would have been technically impossible, as there was no such probe available at the time of the study.

Again, there are no clinical studies proving that there is any benefit of intramuscular epinephrine compared to subcutaneous and intravenous epinephrine. Most clinical guidelines support intramuscular injection with the EAI when required in the treatment of anaphylaxis [18]. The findings of this study and others noted above suggest that the variability in children’s STMD and STBD supports the research and development into EAI with different characteristics including varying needle lengths, pressure required to trigger injection, and total delivered doses. Currently, we believe the only method of confirming that an EAI will deliver epinephrine intramuscularly is by measuring STMD_min_, STMD_max_, STBD_min,_ and STBD_max_ with an ultrasound measurement for each individual patient. If the STBD_max_ is less than the clinically important needle length, then having the patient or assisting adult squeeze the vastus lateralis muscle while giving the EAI may prevent the needle from hitting the femur. It is important that the EAI does not hit the fingers that are squeezing the muscle. If the STMD_max_ or STMD_min_ is greater than the length of the EAI needle minus 2 mm, we believe it is extremely important for patients to always carry two EAI and call for medical assistance immediately if they are required.

## Conclusions

In children with food allergy, the HPEAI currently available in North America will deliver epinephrine to the intramuscular space in approximately 9 out of 10 children when used properly. In a significant number of children weighing 15–30 kg, there is a risk (11–38 %) of injecting the epinephrine into the bone with HPEAI (Epipen Jr^®^, Auvi-Q^®^/Allerject^®^ 0.15 mg and Jext^®^). There is a 9 % risk of a subcutaneous injection in children and adolescents weighing more than 30 kg, using high pressure EAI (Epipen^®^ or Auvi-Q^®^/Allerject^®^ 0.30 mg, Jext^®^), whereas when using a low pressure EAI (Emerade^®^) we found a 2 % risk of subcutaneous injection.

## Additional file

10.1186/s13223-016-0110-8 Patient characteristics for subjects at risk of intraosseous and subcutaneous injections with the available epinephrine auto-injectors.

## Abbreviations

EAI: epinephrine auto-injector
HPEAI: high pressure EAI
LPEAI: low pressure EAI
STMD: skin to muscle distance
STBD: skin to bone distance
STMD_min_: skin to muscle distance. no/minimal pressure
STMD_max_: skin to muscle distance. high pressure. 8 lb
STBD_min_: skin to bone distance. no/minimal pressure
STBD_max_: skin to bone distance. high pressure. 8 lb
