# Erratum to: Do epinephrine auto-injectors have an unsuitable needle length in children and adolescents at risk for anaphylaxis from food allergy?

**DOI:** 10.1186/s13223-017-0205-x

**Published:** 2017-07-07

**Authors:** Sten Dreborg, Xia Wen, Laura Kim, Gina Tsai, Immaculate Nevis, Ryan Potts, Jack Chiu, Arunmozhi Dominic, Harold Kim

**Affiliations:** 1 Women’s and Children’s Health, University of Uppsala, Uppsala, Sweden; 2 Faculty of Science, McGill University, Montreal, Canada; 3 Faculty of Medicine, University British Columbia, Vancouver, Canada; 4 Goodman School of Business, Brock University, St. Catharines, Canada; 5 Farncombe Family Digestive Health Unit, McMaster University, Hamilton, Canada; 6 Department of Medicine, Western University, London, Canada; 7 Department of Medicine, McMaster University, Hamilton, ON Canada

## Erratum to: Allergy Asthma Clin Immunol (2016) 12:11 DOI 10.1186/s13223-016-0110-8

After publication of the article [1] it was brought to our attention that the incorrect length of needle was used for one of the epinephrine auto-injectors (EAI), Jext^®^. The length of the needle of the Jext^®^ 0.15 mg is not 15.7 mm as mentioned in the paper, but 13 mm. After correcting the needle length for Jext^®^, the results of the study do not change aside for those involving Jext^®^. The results of the primary outcome variable for Jext^®^ are now the same as the other high pressure EAIs (HPEAIs) and do not lead to intra-osseous injections 38% but 11% between 15 and 30 kg. This is the same risk as the Epipen^®^ and the Auvi-Q^®^.
