# Supplementary material for: Do epinephrine auto-injectors have an unsuitable needle length in children and adolescents at risk for anaphylaxis from food allergy?
Source: Allergy Asthma Clin Immunol. 2016 Mar 6;12:11. doi: 10.1186/s13223-016-0110-8 (PMC4779571; doi:10.1186/s13223-016-0110-8)
Supplement: Supplementary file 1 — 10.1186/s13223-016-0110-8 Patient characteristics for subjects at risk of intraosseous and subcutaneous injections with the available epinephrine auto-injectors. [file 13223_2016_110_MOESM1_ESM.docx]

**Table S1a**

Children 15-30 kg

Characteristics of 11 children at risk of injection of epinephrine into bone or the interosseous space if using an EAI with 12.7 mm total needle length STBDmax < 12.7 (Epipen Jr^®^/Auvi-Q^®^/Allerject^®^).

| **Age mo** | **Sex** | **Height cm** | **Weight kg** | **BMI** | **STMD _min_** | **STMD _max_** | **STBD _min_** | **STBD _max_** | **Allergic diseases** | | | |  | **Type of allergy** | | | | | | | | |
| --- | --- | --- | --- | --- | --- | --- | --- | --- | --- | --- | --- | --- | --- | --- | --- | --- | --- | --- | --- | --- | --- | --- |
|  |  |  |  |  |  |  |  |  | **Rhi-nitis** | | **Asthma** | **Ecze-ma** |  | Egg milk | Wheat  soy  etc. | Peanut | | Tree  nuts | | Pollen  fruits | | Indoor  allergens |
| 108 |  | 142.2 | 28.6 | 14.1 | 4.7 | 4.6 | 28.1 | 11.7 | 1 | 1 | | 1 |  | 0 | 0 | 1 | | 1 | | 0 | | 0 |
| 48 |  | 109.2 | 19.3 | 16.2 | 6.1 | 5.6 | 27.5 | 12.4 | 0 | 1 | | 1 |  | 0 | 0 | 1 | | 0 | | 0 | | 0 |
| 120 |  | 134.6 | 23.1 | 12.8 | 2.7 | 2.3 | 24.6 | 11.1 | 0 | 1 | | 0 |  | 1 | 0 | 1 | | 0 | | 0 | | 0 |
| 48 |  | 106.7 | 15.0 | 13.2 | 4 | 3.5 | 24.7 | 9.8 | 0 | 0 | | 0 |  | 1 | 1 | 1 | | 0 | | 0 | | 0 |
| 72 |  | 124.5 | 24.0 | 15.5 | 4.7 | 4.6 | 30.1 | 10.1 | 0 | 0 | | 0 |  | 1 | 0 | 0 | | 0 | | 0 | | 0 |
| 48 | F | 104.1 | 15.4 | 14.2 | 6.4 | 6 | 25 | 11.8 | 0 | 1 | | 0 |  | 0 | 0 | 0 | | 1 | | 0 | | 0 |
| 48 | F | 106.7 | 17.7 | 15.5 | 6.1 | 5.1 | 26.9 | 11.7 | 0 | 0 | | 1 |  | 0 | 0 | 1 | | 0 | | 0 | | 0 |
| 72 |  | 144.8 | 22.7 | 10.8 | 6.3 | 5.5 | 21 | 12.4 | 1 | 1 | | 1 |  | 1 | 0 | 0 | | 0 | | 0 | | 0 |
| 60 | F | 106.7 | 15.9 | 13.9 | 5.4 | 4.7 | 25.7 | 12.6 | 1 | 1 | | 0 |  | 0 | 0 | 1 | | 1 | | 0 | | 0 |
| 48 |  | 106.7 | 15.4 | 13.6 | 6.6 | 5.7 | 23.4 | 12.3 | 1 | 1 | | 0 |  | 1 | 1 | 0 | | 0 | | 0 | | 0 |
| 84 |  | 116.8 | 19.5 | 14.3 | 7.1 | 5.3 | 22.1 | 12.1 | 0 | 1 | | 0 |  | 0 | 0 | 1 | | 0 | | 0 | | 0 |
| **n** | 3F |  |  |  |  |  |  |  | 4 | $8$ | | $4$ |  | $5$ | 2 | 7 | | 3 | | 0 | | $0$ |
| **Median** | | 109.2 | 19.3 | 14.1 | 6.1 | 5.1 | 25.0 | 11.8 |  |  | |  |  |  |  | |  | |  | |  |  |
| **Max** |  | 148.8 | 28.6 | 16.2 | 7.1 | 6.0 | 30.1 | 12.6 |  |  | |  |  |  |  | |  | |  | |  |  |
| **Min** |  | 104.1 | 15.0 | 10.8 | 2.7 | 2.3 | 21.0 | 9.8 |  |  | |  |  |  |  | |  | |  | |  |  |

**Table S1b**

Children 15-30 kg

Characteristics of 38 children at risk of injection of epinephrine into the bone or the interosseous space if using a high pressure EAI with 15.7 mm total needle length (Jext^®^).

|  | **Age** | **Sex** | **Height cm** | **Weight kg** | **BMI** | **STMD**  **_min_** | **STMD**  **_max_** | **STBD**  **_min_** | **STBD**  **_max_** | **Allergic diseases** | | | |  | **Type of allergy** | | | | | | |
| --- | --- | --- | --- | --- | --- | --- | --- | --- | --- | --- | --- | --- | --- | --- | --- | --- | --- | --- | --- | --- | --- |
|  |  |  |  |  |  |  |  |  |  | **Rhi-nitis** | **Asthma** | **Ecze-ma** | **Urti-caria** |  | **Egg. milk wheatssoy** | **Lentils**  **beans etc.** | **Shrimp fish**  **etc.** | **Pea-nut** | **Tree- nuts** | **Pollen**  **fruits**  **vege-tables** | **Indoor** |
|  | 4 |  | 111.8 | 16.1 | 12.9 | 5.1 | 4.6 | 22.1 | 15.5 | 0 | 1 | 0 | 0 |  | 0 | 0 | 0 | 1 | **0** | **0** | **0** |
|  | 4 |  | 109.2 | 18.1 | 15.2 | 7.3 | 5.9 | 28.2 | 15.6 | 1 | 1 | 1 | 0 |  | 0 | 1 | 0 | 0 | 1 | 0 | 1 |
|  | 6 |  | 91.4 | 14.5 | 17.4 | 3 | 2.6 | 23.2 | 15.0 | 0 | 0 | 0 | 1 |  | 1 | 0 | 1 | 0 | 0 | 0 | 0 |
|  | 4 | F | 86.4 | 18.1 | 24.3 | 7.2 | 7.1 | 30.4 | 15.2 | 0 | 1 | 1 | 1 |  | 0 | 0 | 0 | 1 | 0 | 0 | 0 |
|  | 5 |  | 106.7 | 18.1 | 15.9 | 4.4 | 4.1 | 26.2 | 13.6 | 0 | 1 | 1 | 1 |  | 0 | 0 | 0 | 1 | 0 | 1 | 1 |
|  | 7 |  | 121.9 | 22.9 | 15.4 | 5.8 | 4.7 | 29.1 | 15.3 | 0 | 0 | 1 | 0 |  | 0 | 0 | 0 | 0 | 1 | 0 | 0 |
|  | 4 |  | 101.6 | 15.9 | 15.4 | 5.7 | 5.1 | 25.7 | 14.3 | 0 | 0 | 1 | 0 |  | 1 | 0 | 0 | 0 | 1 | 0 | 0 |
|  | 6 |  | 121.9 | 21.8 | 14.6 | 5.9 | 5.6 | 30.2 | 14.8 | 1 | 1 | 0 | 1 |  | 1 | 0 | 0 | 1 | 1 | 0 | 0 |
|  | 9 |  | 142.2 | 28.6 | 14.1 | 4.7 | 4.6 | 28.1 | 11.7 | 1 | 1 | 1 | 0 |  | 0 | 0 | 0 | 1 | 1 | 1 | 1 |
|  | 3 | F | 104.1 | 16.3 | 15.1 | 5.9 | 5.7 | 24.7 | 13.1 | 0 | 0 | 0 | 0 |  | 1 | 0 | 0 | 1 | 0 | 0 | 0 |
|  | 4 |  | 109.2 | 19.3 | 16.2 | 6.1 | 5.6 | 27.5 | 12.4 | 0 | 1 | 1 | 0 |  | 0 | 0 | 0 | 1 | 0 | 1 | 0 |
|  | 10 |  | 134.6 | 23.1 | 12.8 | 2.7 | 2.3 | 24.6 | 11.1 | 0 | 1 | 0 | 0 |  | 1 | 0 | 0 | 1 | 0 | 0 | 0 |
|  | 4 |  | 109.2 | 18.6 | 15.6 | 7.4 | 5.3 | 32.8 | 14.9 | 0 | 0 | 0 | 0 |  | 1 | 0 | 0 | 1 | 0 | 0 | 0 |
|  | 5 |  | 111.8 | 20.4 | 16.3 | 5.8 | 5.3 | 31.7 | 14.6 | 0 | 0 | 0 | 0 |  | 0 | 0 | 0 | 1 | 1 | 0 | 0 |
|  | 6 |  | 119.4 | 20.0 | 14.0 | 6 | 5.1 | 27.3 | 13.5 | 1 | 0 | 0 | 0 |  | 1 | 0 | 0 | 0 | 0 | 0 | 1 |
|  | 4 |  | 106.7 | 15.0 | 13.2 | 4 | 3.5 | 24.7 | 9.8 | 0 | 0 | 0 | 0 |  | 1 | 0 | 0 | 1 | 0 | 1 | 1 |
|  | 6 |  | 101.6 | 20.0 | 19.3 | 5.7 | 5.2 | 24.6 | 13.8 | 1 | 0 | 0 | 0 |  | 1 | 0 | 0 | 1 | 0 | 1 | 1 |
|  | 5 |  | 114.3 | 28.1 | 21.5 | 4.6 | 4.5 | 30.6 | 13.9 | 0 | 1 | 0 | 0 |  | 0 | 0 | 0 | 0 | 1 | 1 | 1 |
|  | 6 |  | 124.5 | 24.0 | 15.5 | 4.7 | 4.6 | 30.1 | 10.1 | 0 | 0 | 0 | 0 |  | 1 | 0 | 0 | 0 | 0 | 0 | 0 |
|  | 6 |  | 111.8 | 16.8 | 13.4 | 6.6 | 4.8 | 29.8 | 13.5 | 0 | 1 | 0 | 0 |  | 0 | 0 | 0 | 1 | 0 | 0 | 1 |
|  | 4 | F | 104.1 | 15.4 | 14.2 | 6.4 | 6 | 25 | 11.8 | 0 | 1 | 0 | 0 |  | 0 | 0 | 0 | 0 | 1 | 0 | 0 |
|  | 4 | F | 106.7 | 17.7 | 15.5 | 6.1 | 5.1 | 26.9 | 11.7 | 0 | 0 | 1 | 1 |  | 0 | 0 | 0 | 1 | 0 | 0 | 0 |
|  | 5 |  | 112.0 | 20.4 | 16.3 | 8.9 | 6.2 | 34 | 14.6 | 1 | 0 | 1 | 0 |  | 0 | 0 | 0 | 1 | 1 | 0 | 0 |
|  | 6 |  | 109.2 | 17.2 | 14.4 | 4.3 | 3.5 | 24.2 | 13.0 | 0 | 0 | 0 | 0 |  | 1 | 0 | 0 | 0 | 0 | 0 | 0 |
|  | 6 |  | 116.8 | 20.0 | 14.6 | 5.3 | 5.3 | 24.7 | 15.5 | 1 | 1 | 0 | 0 |  | 0 | 0 | 0 | 1 | 1 | 1 | 0 |
|  | 2 |  | 83.8 | 16.3 | 23.2 | 9.3 | 7.1 | 24.3 | 13.6 | 0 | 1 | 0 | 0 |  | 1 | 0 | 0 | 0 | 0 | 0 | 0 |
|  | 5 |  | 111.8 | 18.4 | 14.7 | 5 | 4.1 | 27.3 | 14.2 | 0 | 0 | 1 | 0 |  | 0 | 0 | 0 | 1 | 1 | 0 | 0 |
|  | 6 |  | 144.8 | 22.7 | 10.8 | 6.3 | 5.5 | 21 | 12.4 | 1 | 1 | 1 | 0 |  | 1 | 0 | 0 | 0 | 0 | 1 | 0 |
|  | 5 |  | 114.3 | 20.9 | 16.0 | 4.9 | 3.9 | 23.8 | 13.6 | 0 | 1 | 0 | 0 |  | 0 | 0 | 0 | 1 | 0 | 0 | 0 |
|  | 5 | F | 106.7 | 15.9 | 13.9 | 5.4 | 4.7 | 25.7 | 12.6 | 1 | 1 | 0 | 0 |  | 0 | 0 | 0 | 1 | 1 | 0 | 0 |
|  | 3 |  | 99.1 | 16.3 | 16.6 | 8.2 | 7.5 | 23.6 | 15.6 | 0 | 0 | 0 | 0 |  | 0 | 0 | 0 | 1 | 1 | 0 | 0 |
|  | 4 |  | 106.7 | 15.4 | 13.6 | 6.6 | 5.7 | 23.4 | 12.3 | 1 | 1 | 0 | 0 |  | 1 | 1 | 0 | 0 | 0 | 0 | 1 |
|  | 6 | F | 116.8 | 18.1 | 13.3 | 5.3 | 4.4 | 32.1 | 15.3 | 0 | 0 | 0 | 0 |  | 0 | 0 | 0 | 0 | 1 | 1 | 1 |
|  | 7 |  | 116.8 | 19.5 | 14.3 | 7.1 | 5.3 | 22.1 | 12.1 | 0 | 1 | 0 | 0 |  | 0 | 0 | 0 | 1 | 0 | 0 | 0 |
|  | 5 | F | 108.0 | 17.7 | 15.2 | 6.6 | 5.8 | 22.1 | 13.1 | 1 | 0 | 0 | 0 |  | 0 | 0 | 0 | 0 | 0 | 1 | 1 |
|  | 8 | F | 129.5 | 26.3 | 15.7 | 5.5 | 4.6 | 27.6 | 13.9 | 0 | 0 | 0 | 0 |  | 0 | 0 | 0 | 0 | 1 | 1 | 0 |
|  | 6 |  | 119.4 | 22.7 | 15.9 | 5.8 | 4.8 | 33.8 | 14.9 | 0 | 0 | 0 | 0 |  | 0 | 0 | 0 | 1 | 0 | 0 | 0 |
|  | 7 |  | 119.4 | 19.5 | 13.7 | 3.7 | 3.1 | 29 | 13.6 | 0 | 0 | 0 | 0 |  | 0 | 0 | 0 | 0 | 0 | 1 | 0 |
| **n** | 8 | 8F |  |  |  |  |  |  |  | 11 | 18 | 11 | 5 |  | 144 | 2 | 1 | 22 | 15 | 12 | 12 |
| **Mean** | 5.7 |  | 115.6 | 18.9 | 14.7 | 5.7 | 5.1 | 24.5 | 13.6 |  |  |  |  |  |  |  |  |  | | | |
| **max** | 8.0 |  | 144.8 | 26.3 | 23.2 | 9.3 | 7.5 | 33.8 | 15.6 |  |  |  |  |  |  |  |  |  | | | |
| **min** | 2.0 |  | 83.8 | 15.4 | 10.8 | 3.7 | 3.1 | 21.0 | 9.8 |  |  |  |  |  |  |  |  |  | | | |

**Table S1c**

Children and adolescents > 30 kg

Characteristics of 3children at risk of injection of epinephrine into bone or the interosseous space if using an EAI with 15.2 or 15.7 mm total needle length (Epipen^®^/Auvi-Q^®^/Allerject^®^ and Jext, respectively).

|  |  |  |  |  |  |  |  |  |  | **Allergic diseases** | | |  | **Allergy** | | | | | | | | | | | |
| --- | --- | --- | --- | --- | --- | --- | --- | --- | --- | --- | --- | --- | --- | --- | --- | --- | --- | --- | --- | --- | --- | --- | --- | --- | --- |
|  | **Age**  **yrs** | **Sex** | **Height**  **cm** | **Weight**  **kg** | **BMI** | **STMD min** | **STMD max** | **STBD min** | **STBD max** | **Rhinitis** | **Asthma** | **Eczema** |  | **Egg milk wheat soy** | **Lentils beans etc.** | **Shrimp fish etc.** | | **Pea-nut** | | **Tree**  **nuts** | | **Pollen fruits vege-tables** | | **In-door** | |
|  | 12 | M | 142.2 | 33.1 | 16.4 | 4.9 | 4.8 | 34.8 | **13.9** | 0 | 0 | 0 |  | 0 | 0 | 0 | | 1 | | 0 | | 1 | | 1 | |
|  | 7 | M | 144.8 | 31.3 | 14.9 | 5.9 | 4.5 | 32.1 | **13.9** | 1 | 0 | 0 |  | 0 | 0 | 0 | | 0 | | 1 | | 0 | | 1 | |
|  | 12 | M | 137.2 | 44.9 | 23.9 | 5.4 | 4.6 | 23.5 | **12.8** | 1 | 1 | 0 |  | 0 | 0 | 0 | | 1 | | 0 | | 1 | | 1 | |
| n |  |  |  |  |  |  |  |  |  | 2 | 1 | 0 |  | 0 | 0 | 0 | | 2 | | 1 | | 2 | | 3 | |
| Mean | 10,3 | 0 F | 141.4 | 36.4 | 18.1 | 5.4 | 4.8 | 30.1 | 13.5 |  |  |  |  |  |  |  | |  | |  | |  | |  | |
| max | 12 |  | 144.8 | 88.9 | 23.7 | 5.9 | 4.8 | 34.8 | 13.9 |  |  |  |  |  |  |  | |  | |  | |  | |  | |
| min | 7 |  | 137.2 | 31.3 | 14.9 | 4.9 | 4.5 | 23.5 | 12.8 |  |  |  |  |  |  | |  | |  | |  | |  | |  |

**Table S1d**

Children and adolescents >30 kg

Characteristics of 9 children and adolescents at risk of injection of epinephrine into the subcutaneous tissue instead of the muscle if using an EAI with 12.7 mm needle applied with high pressure. i.e. the STMD_max_ > 10.7 mm (Epipen^®^/Auvi-Q^®^/Allerject^®^).

|  |  |  |  |  |  | **Skin to muscle/bone distance** | | | |  | **Allergic disease** | | | |  | **Type of allergy** | | | | | | |
| --- | --- | --- | --- | --- | --- | --- | --- | --- | --- | --- | --- | --- | --- | --- | --- | --- | --- | --- | --- | --- | --- | --- |
|  | **Age** | **Sex** | **Height cm** | **Weight kg** | **BMI** | **STMDmin mm** | **STMD max mm** | **STBD min mm** | **STBD max mm** |  | **Rhinitis** | **Asthma** | **Ecze-ma** | **Urti-caria** |  | **Egg milk wheat soy** | **Lentils beans etc.** | **Shrimp fish** | **Pea-nut** | **Tree-nuts** | **Pollens fruits** | **Pets mites molds** |
|  | 11 | F | 144.8 | 30.8 | 15 | 6.7 | 6.6 | 3.3 | 20.4 |  | 1 | 0 | 0 | 0 |  |  |  |  | 1 |  |  |  |
|  | 11 | F | 147.3 | 63.0 | 29 | 15.1 | 14.8 | 49.6 | 32.8 |  | 1 | 1 | 0 | 0 |  |  |  |  | 1 |  |  |  |
|  | 11 | F | 157.5 | 75.7 | 31 | 18.5 | 15.8 | 55.0 | 38.7 |  | 0 | 0 | 0 | 0 |  |  |  |  | 1 |  |  | 1 |
|  | 17 | F | 174.0 | 75.3 | 25 | 15.8 | 14.1 | 58.6 | 38.5 |  | 1 | 1 | 1 | 0 |  |  |  |  |  | 1 | 1 | 1 |
|  | 14 |  | 170.2 | 88.9 | 31 | 15.4 | 14.4 | 56.9 | 39.4 |  | 1 | 0 | 0 |  |  |  |  | 1 |  | 1 | 1 | 1 |
|  | 16 | F | 162.6 | 72.6 | 27 | 26.1 | 18.5 | 60.4 | 41.2 |  | 1 | 1 | 0 | 0 |  |  |  |  | 1 |  | 1 | 1 |
|  | 16 | F | 172.7 | 83.5 | 28 | 23.8 | 20.2 | 63.8 | 39.3 |  | 0 | 0 | 0 | 0 |  | 1 |  |  |  |  |  |  |
|  | 18 | F | 160.0 | 59.0 | 23 | 18.3 | 17.3 | 64.3 | 56.0 |  | 0 | 0 | 1 | 0 |  |  | 1 |  |  |  | 1 | 1 |
|  | 13 | F | 162.6 | 72.1 | 27 | 19.0 | 15.3 | 52.3 | 29.6 |  | 1 | 1 | 0 | 0 |  |  |  |  | 1 |  | 1 | 1 |
| **Sum** |  | 8 F |  |  |  |  |  |  |  |  | 6 | 4 | 2 | 0 |  | 1 | 1 | 1 | 5 | 2 | 5 | 6 |
| **Mean** | 14.1 |  | 161.3 | 69.0 | 26 | 17.6 | 15.2 | 55.2 | 37.3 |  |  |  |  |  |  |  |  |  |  |  |  |  |
| **max** | 18.0 |  | 174.0 | 88.9 | 31 | 26.1 | 20.2 | 64.3 | 56.0 |  |  |  |  |  |  |  |  |  |  |  |  |  |
| **min** | 11.0 |  | 147.3 | 59.0 | 23 | 15.1 | 14.1 | 49.6 | 29.6 |  |  |  |  |  |  |  |  |  |  |  |  |  |
| **BMI > 20** |  |  |  |  | 7/9 |  |  |  |  |  |  |  |  |  |  |  |  |  |  |  |  |  |
| **BMI > 25** |  |  |  |  | 6/9 |  |  |  |  |  |  |  |  |  |  |  |  |  |  |  |  |  |

**Table S1e**

Children and adolescents >30 kg

Characteristics of 9 teenagers at risk of injection of epinephrine subcutaneously if using an EAI with 15.2 or 15.7 mm total needle length (Epipen^®^/Auvi-Q^®^/Allerject^®^ and Jext. Respectively). i.e. 13.2 and 13.7 mm from the skin surface to the inner surface of the fascia lata the distance measured by STMD_max_.

|  | **Age** | **Sex** | **Height cm** | **Weight (kg)** | **BMI** | **Allergic diseases** | | |  | **Skin to bone and muscle dist.** | | | | | | |  | **Allergy-sensitization** | | | | | |  |  |
| --- | --- | --- | --- | --- | --- | --- | --- | --- | --- | --- | --- | --- | --- | --- | --- | --- | --- | --- | --- | --- | --- | --- | --- | --- | --- |
|  |  |  |  |  |  | **Rhi-nitis** | **Asthma** | **Ecze-ma** |  | **STMD _min_** | **STMD _max_** | | **STBD _min_** | | **STBD _max_** | |  | **Milk egg**  **wheat** | **Lentils soy** | **Shrimp fish** | **Pea- nuts** | **Tree nuts** | **Pollens fruits** | **Pets mites molds** |  |
|  | 12 |  | 168 | 79..4 | 28.2 | 1 | 1 | 0 |  | 17.3 | | 16.5 | | 46.8 | | 33.9 |  | 0 | 0 | 0 | 1 | 1 | 1 | 1 |  |
|  | 11 | F | 147 | 63.0 | 29.1 | 1 | 1 | 0 |  | 15.1 | | 14.8 | | 49.6 | | 32.8 |  | 0 | 0 | 0 | 1 | 0 | 0 | 1 |  |
|  | 11 | F | 158 | 75.7 | 30.5 | 0 | 0 | 0 |  | 18.5 | | 15.8 | | 55.0 | | 38.7 |  | 0 | 0 | 0 | 1 | 0 | 0 | 0 |  |
|  | 17 | F | 174 | 75.3 | 24.9 | 1 | 1 | 1 |  | 15.8 | | 14.1 | | 58.6 | | 38.5 |  | 0 | 0 | 0 | 0 | 1 | 0 | 0 |  |
|  | 14 |  | 170 | 88.9 | 30.7 | 1 | 0 | 0 |  | 15.4 | | 14.4 | | 56.9 | | 39.4 |  | 0 | 0 | 1 | 0 | 1 | 0 | 0 |  |
|  | 16 | F | 163 | 72.6 | 27.5 | 1 | 1 | 0 |  | 26.1 | | 18.5 | | 60.4 | | 41.2 |  | 0 | 0 | 0 | 1 | 0 | 0 | 0 |  |
|  | 16 | F | 173 | 83.5 | 28.0 | 0 | 0 | 0 |  | 23.8 | | 20.2 | | 63.8 | | 39.3 |  | 1 | 0 | 0 | 0 | 0 | 0 | 0 |  |
|  | 18 | F | 160 | 59.0 | 23.0 | 0 | 0 | 1 |  | 18.3 | | 17.3 | | 64.3 | | 56.0 |  | 0 | 1 | 0 | 0 | 0 | 0 | 0 |  |
|  | 13 | F | 163 | 72.1 | 27.3 | 1 | 0 | 0 |  | 19 | | 15.3 | | 52.3 | | 29.6 |  | 0 | 0 | 0 | 1 | 0 | 0 | 0 |  |
| Sum |  | 7F |  |  |  | 6 | 4 | 2 |  |  | |  | |  | |  |  | 1 | 1 | 1 | 5 | 3 | 1 | 2 |  |
| Mean | 14 |  | 163 | 75.3 | 28.0 |  |  |  |  | 18.3 | | 15.8 | | 56.9 | | 38.7 |  |  |  |  |  |  |  |  |  |
| max | 18 |  | 174 | 88.9 | 30.7 |  |  |  |  | 26.1 | | 20.2 | | 64.3 | | 56.0 |  |  |  |  |  |  |  |  |  |
| min | 11 |  | 147 | 59.0 | 23.0 |  |  |  |  | 15.1 | | 14.1 | | 46.8 | | 29.6 |  |  |  |  |  |  |  |  |  |
